# Trends in pneumonia mortality among US adults, 1999 to 2023: A CDC WONDER database analysis

**DOI:** 10.1097/MD.0000000000050389

**Published:** 2026-08-21

**Authors:** Nawei Song, Juan He, Xianghua Shuai, Hana Zhu, Keran Xia

**Affiliations:** a Department of Pediatrics, Qiaosi Branch, The First People’s Hospital of Linping District, Hangzhou, China.

**Keywords:** CDC WONDER, mortality, pneumonia, US adults

## Abstract

Influenza combined with pneumonia creates a persistent public health burden across the United States, continuously threatening adult survival and consuming massive medical resources. This population-based epidemiological research aims to map long-term mortality shifting patterns linked to flu and pneumonia among American adults spanning the 25-year window from 1999 to 2023. Our analytical data were extracted from the Centers for Disease Control and Prevention WONDER public mortality repository. Deaths triggered primarily by influenza or pneumonia were screened through International Classification of Diseases, 10th Revision diagnostic codes J09 to J18. Two core mortality indicators, crude rates and age-standardized mortality rates, were computed for the whole population and separate subgroups. Joinpoint regression modeling was adopted to quantify yearly percentage shifts (annual percent change) and average annual changing magnitudes (average annual percent change), paired with 95% confidence intervals (CIs) to judge statistical significance. Stratified subgroup comparisons were completed across demographic and geographic dimensions, with statistical significance defined as *P* < .05. Across the full research timeframe, the national age-adjusted mortality rate for influenza and pneumonia dropped substantially, falling from 35.93 per 100,000 residents in 1999 to 16.46 per 100,000 residents in 2023, with an average annual decline of 3.18% (95% CI = −3.53 to −2.83, *P* < .05). Mortality burdens consistently ran higher in male populations relative to females. Among all racial subgroups, non-Hispanic Black adults bore the heaviest baseline mortality pressure in 1999 (38.66 per 100,000 population) and also saw the most marked relative reduction by 2023 (19.01 per 100,000 population). Rural counties maintained elevated age-adjusted mortality values compared with metropolitan regions in all survey years. Adults aged 85 and above exhibited the highest crude death rate, peaking at 751.78 per 100,000 people in 1999. Notably, the 55 to 64 middle-aged group was the only stratum with a statistically meaningful upward trend in crude mortality, with an average annual percent change of 0.78% (95% CI = 0.20–1.37, *P* < .05). This study demonstrates long-term declining trends in US influenza and pneumonia-related mortality with persistent disparities by sex, age, race/ethnicity, and urban–rural status.

## 1. Introduction

Influenza and pneumonia form a paired respiratory hazard that imposes enduring public health pressure within the United States, generating extensive annual cases, premature deaths, and overloading local medical service systems year after year.^[1–3]^ Influenza viral infection often weakens lower respiratory tract defenses, raising the risk of secondary pneumonia development; this close clinical correlation explains why the 2 diseases are frequently documented together on official death certification forms.^[4,5]^ According to surveillance reports released by the US Centers for Disease Control and Prevention (CDC), influenza and pneumonia together account for a large share of annual fatalities nationwide, with pneumonia most commonly recorded as the primary underlying cause of mortality.^[6,7]^ Given their tight clinical linkage and co-occurrence in mortality records, aggregating deaths coded under International Classification of Diseases, 10th Revision (ICD-10) influenza and pneumonia categories represents a rational method to track population-level mortality trajectories.

Over the past 3 decades, nationwide death patterns associated with these 2 respiratory disorders have undergone notable transformations. Even so, existing published literature lacks comprehensive long-term trend assessments and in-depth exploration of health inequities across diverse population subgroups. The CDC WONDER platform delivers standardized, anonymized national mortality datasets covering multi-decade time spans, offering reliable population-level evidence for respiratory disease epidemiological analysis. By integrating this database with joinpoint regression tools to pinpoint inflection points in mortality trends, the present research fills 2 major research voids identified in prior work. First, most earlier analyses covered only short time horizons and failed to capture mortality fluctuations occurring after the COVID-19 outbreak in 2020. Second, few previous papers carried out complete stratified analyses covering age brackets, gender, ethnic identity, urban–rural residence, and geographic census regions, leaving subgroup-specific health disparities insufficiently characterized. Findings generated from this research can supply quantitative evidence for customized public health policy design, narrow avoidable health inequalities, and optimize clinical and population-level prevention strategies for high-risk demographic groups.

## 2. Materials and methods

### 2.1. Study data source

This retrospective population-based study used mortality data from the CDC WONDER Underlying Cause of Death database (https://wonder.cdc.gov/), which provides de-identified data on all deaths in the United States with standardized cause-of-death coding. Data were extracted for the period from 1999 to 2023, in line with the availability of ICD-10 coding for influenza and pneumonia.

### 2.2. Study subjects and inclusion/exclusion criteria

Inclusion criteria were: deaths occurring in the United States from 1999 to 2023; influenza and/or pneumonia recorded as the underlying cause of death on death certificates, identified via ICD-10 codes J09 to J18 (J09: influenza due to novel influenza A virus; J10: influenza due to other identified influenza A virus; J11: influenza due to unidentified influenza virus; J12: viral pneumonia, not elsewhere classified; J13–J15: bacterial pneumonia; J16: pneumonia due to other infectious agents; J17: pneumonia in diseases classified elsewhere; J18: pneumonia, unspecified).

Restricting analyses to the underlying cause of death was chosen to ensure consistency with standard population-level mortality studies, minimize potential overestimation of the mortality burden, and enhance comparability with published literature.

Exclusion criteria were: deaths with incomplete demographic information (sex, age, race/ethnicity, or residential address); deaths in individuals under 25 years of age, as this study focuses on adult mortality trends.

Demographic and regional data were extracted, including sex, race/ethnicity (Hispanic, non-Hispanic (NH) Black, NH White, NH other), age (25–34, 35–44, 45–54, 55–64, 65–74, 75–84, 85+ years), urban–rural classification, US Census Region (Northeast, Midwest, South, West), and state. Urban–rural classification adopted the 2013 National Center for Health Statistics Urban–Rural Classification Scheme,^[8]^ dividing counties into metropolitan (urban) areas (≥50,000 population) and nonmetropolitan (rural) areas (<50,000 population). Census regions were categorized in accordance with United States Census Bureau definitions.^[9]^

### 2.3. Outcome measures and calculation methods

The primary outcome was the influenza and pneumonia-related mortality rate, including the crude mortality rate calculated by dividing the number of influenza and pneumonia-related deaths by the corresponding US population size (per 100,000 people) and the age-adjusted mortality rate (AAMR) standardized using the 2000 United States standard population to eliminate the impact of age structure differences on mortality rate comparisons, with 95% confidence intervals (CIs) calculated to reflect statistical uncertainty.

For age-group analyses, crude mortality rates were used because age strata were mutually exclusive and directly reported by CDC WONDER; age adjustment within narrow age groups is unnecessary and would not meaningfully alter trend interpretation, and crude rates support straightforward interpretation of the absolute mortality burden within each age category.

For urbanization-stratified analyses, 2020 AAMR data were used as a proxy for 2023 due to data availability constraints in the CDC WONDER database. This substitution may limit the interpretation of urban–rural differences after 2020 given changes in rural healthcare access and service availability, and conclusions regarding urban–rural comparisons for 2023 should be interpreted cautiously.

### 2.4. Statistical analysis

All statistical analyses were conducted using R software (version 4.2.3; R Core Team, R Foundation for Statistical Computing). Joinpoint regression models were fitted using the “Joinpoint” package to identify significant temporal changes in mortality trends. A maximum of 3 joinpoints was tested, and model selection was based on the Bayesian Information Criterion and permutation tests to determine the optimal number of statistically significant trend segments. The annual percent change for each trend segment and the average annual percent change (AAPC) for the full study period were estimated with corresponding 95% CIs. Trends were considered statistically significant if 95% CIs for annual percent change excluded zero or *P* < .05 across demographic, geographic, and urban–rural subgroups using stratified analyses.

## 3. Results

### 3.1. Overall influenza and pneumonia-related mortality trends (1999–2023)

From 1999 to 2023, the total number of influenza and pneumonia-related deaths decreased from 63,006 in 1999 to 44,625 in 2023, representing a 29.17% change. The overall AAMR decreased from 35.93 per 100,000 population (95% CI = 35.65–36.21) in 1999 to 16.46 per 100,000 population (95% CI = 16.31–16.61) in 2023. The AAPC was −3.18% (95% CI = −3.53 to −2.83, *P* < .05; Table 1).

**Table 1:** Influenza and pneumonia deaths and AAMR in the United States from 1999 to 2023 and their changing trends.

Characteristic	Deaths			AAMR		
	1999	2023	Percent change (%)	1999 (95% CI)	2023 (95% CI)	AAPC (95% CI)
Total	63,006	44,625	−29.17	35.93 (35.65 to 36.21)	16.46 (16.31 to 16.61)	−3.18 (−3.53 to −2.83)*
Sex						
Female	35,669	21,750	−39.02	31.40 (31.07 to 31.73)	14.17 (13.98 to 14.36)	−3.25 (−3.65 to −2.86)*
Male	27,337	22,875	−16.32	43.69 (43.16 to 44.22)	19.47 (19.21 to 19.72)	−3.27 (−3.59 to −2.95)*
Census region						
Northeast	14,249	9138	−35.87	37.72 (37.10 to 38.34)	18.04 (17.67 to 18.41)	−2.90 (−3.30 to −2.50)*
Midwest	16,120	9075	−43.70	37.65 (37.07 to 38.23)	15.89 (15.56 to 16.22)	−2.99 (−3.42 to −2.56)*
South	22,149	16,929	−23.57	36.56 (36.08 to 37.04)	16.44 (16.19 to 16.69)	−3.14 (−3.44 to −2.83)*
West	10,488	9483	−9.58	30.81 (30.22 to 31.41)	15.55 (15.23 to 15.86)	−2.92 (−5.25 to −0.55)*
Race						
Hispanic	2130	3973	86.53	28.67 (27.40 to 29.93)	14.46 (13.99 to 14.92)	−3.37 (−3.76 to −2.97)*
NH Black	5576	5093	−8.66	38.66 (37.63 to 39.69)	19.01 (18.47 to 19.55)	−2.87 (−3.23 to −2.51)*
NH White	53,943	32,878	−39.05	35.94 (35.64 to 36.25)	16.56 (16.37 to 16.74)	−3.14 (−3.51 to −2.76)*
NH other	1097	2519	129.63	27.99 (26.27 to 29.72)	13.36 (12.83 to 13.88)	−3.78 (−4.59 to −2.96)*
Urbanization†						
Metropolitan	49,540	36,983	−25.34	35.06 (34.75 to 35.37)	19.25 (19.07 to 19.44)	−3.14 (−3.56 to −2.72)*
Nonmetropolitan	13,466	7642	−43.24	39.61 (38.94 to 40.28)	23.49 (23.02 to 23.96)	−2.42 (−2.86 to −1.97)*
Age groups‡						
25–34 yr	339	458	35.10	0.84 (0.75 to 0.93)	1.01 (0.91 to 1.10)	0.63 (−0.75 to 2.03)
35–44 yr	1063	967	−9.03	2.36 (2.22 to 2.50)	2.18 (2.04 to 2.32)	0.04 (−0.80 to 0.89)
45–54 yr	1697	1739	2.47	4.64 (4.42 to 4.86)	4.29 (4.09 to 4.50)	0.26 (−0.50 to 1.03)
55–64 yr	2625	4602	75.31	11.04 (10.62 to 11.46)	11.00 (10.68 to 11.31)	0.78 (0.20 to 1.37)*
65–74 yr	6861	9005	31.25	37.25 (36.37 to 38.13)	25.96 (25.43 to 26.50)	−1.40 (−2.22 to −0.58)*
75–84 yr	19,192	12,623	−34.23	156.99 (154.77 to 159.21)	68.72 (67.52 to 69.92)	−3.42 (−3.74 to −3.10)*
85+ yr	31,229	15,231	−51.23	751.78 (743.44 to 760.12)	245.86 (241.96 to 249.76)	−4.50 (−7.58 to −1.31)*

AAMR = age-adjusted mortality rate, AAPC = average annual percent change, CI = confidence interval, NH = non-Hispanic.

† In the context of urbanization, the 2023 AAMR data were substituted with that from 2020, and the AAPC was calculated based on the period from 1999 to 2020.

‡ For the age groups, the crude mortality rate was used as a substitute for AAMR, and the AAPC was computed based on the crude mortality rate.

* *P* < .05.

### 3.2. Mortality trends by sex

**Figure 1.**
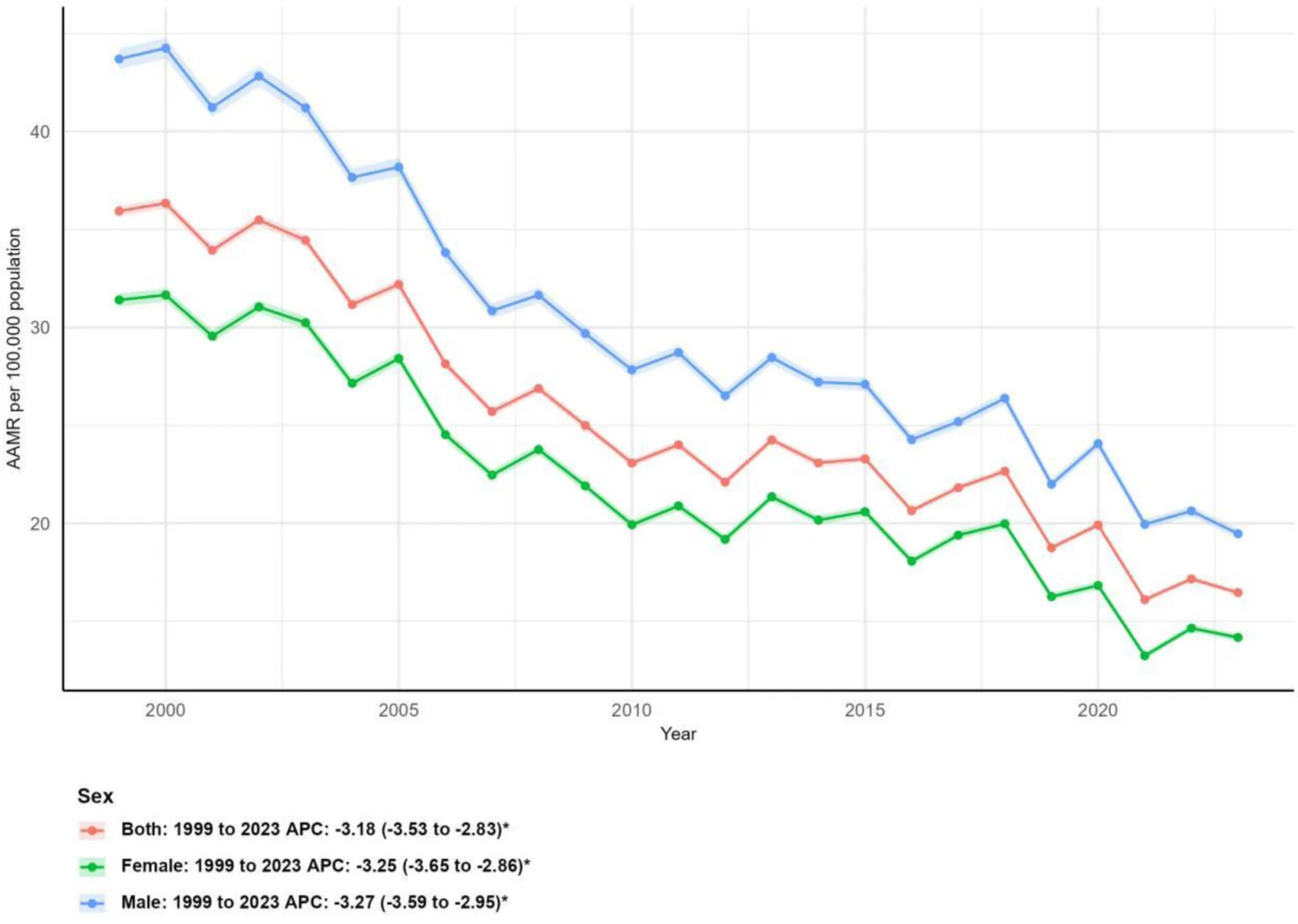
Mortality trends by sex. AAMR = age-adjusted mortality rate, APC = annual percent change.

In 1999, the AAMR was 43.69 per 100,000 population for males and 31.40 per 100,000 population for females. In 2023, the AAMR was 19.47 per 100,000 population for males and 14.17 per 100,000 population for females. The AAPC was −3.25% (95% CI = −3.65 to −2.86) for females and −3.27% (95% CI = −3.59 to −2.95) for males (both *P* < .05). The total number of deaths decreased by 39.02% in females and 16.32% in males over the study period (Fig. 1).

### 3.3. Mortality trends by race/ethnicity

**Figure 2.**
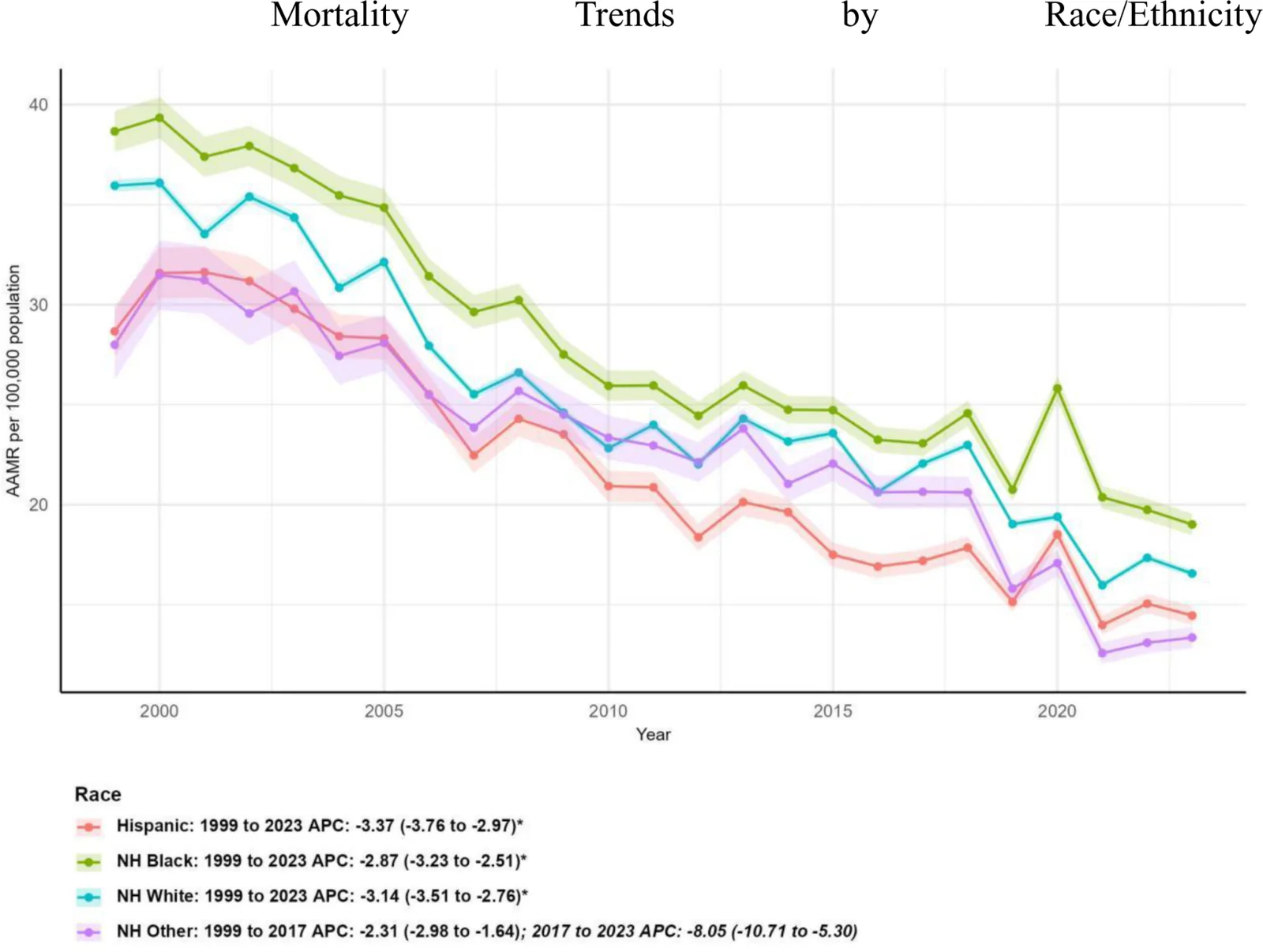
Mortality trends by race/ethnicity. AAMR = age-adjusted mortality rate, APC = annual percent change, NH = non-Hispanic.

In 1999, the AAMR was highest among NH Black individuals (38.66 per 100,000 population), followed by NH White individuals (35.94 per 100,000 population), Hispanic individuals (28.67 per 100,000 population), and NH other individuals (27.99 per 100,000 population). By 2023, all groups exhibited decreased AAMR: 19.01, 16.56, 14.46, and 13.36 per 100,000 population for NH Black, NH White, Hispanic, and NH other individuals, respectively (Fig. 2).

### 3.4. Mortality trends by census region

**Figure 3.**
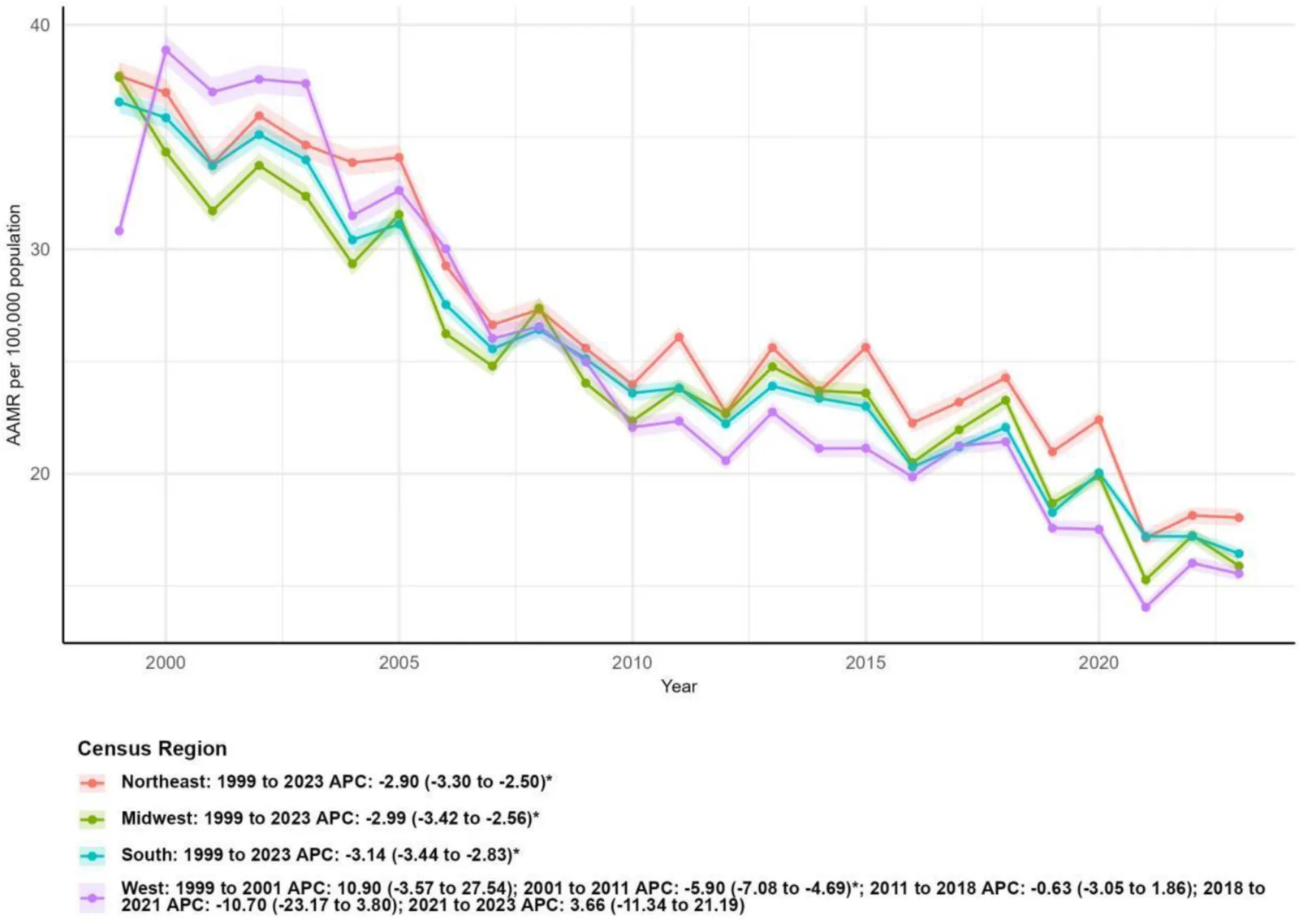
Mortality trends by census region. AAMR = age-adjusted mortality rate, APC = annual percent change.

In 1999, the AAMR was highest in the Northeast (37.72 per 100,000 population) and Midwest (37.65 per 100,000 population), followed by the South (36.56 per 100,000 population) and West (30.81 per 100,000 population). In 2023, the AAMR was lowest in the West (15.55 per 100,000 population), followed by the Midwest (15.89 per 100,000 population), South (16.44 per 100,000 population), and Northeast (18.04 per 100,000 population; Fig. 3).

### 3.5. Mortality trends by urbanization level

**Figure 4.**
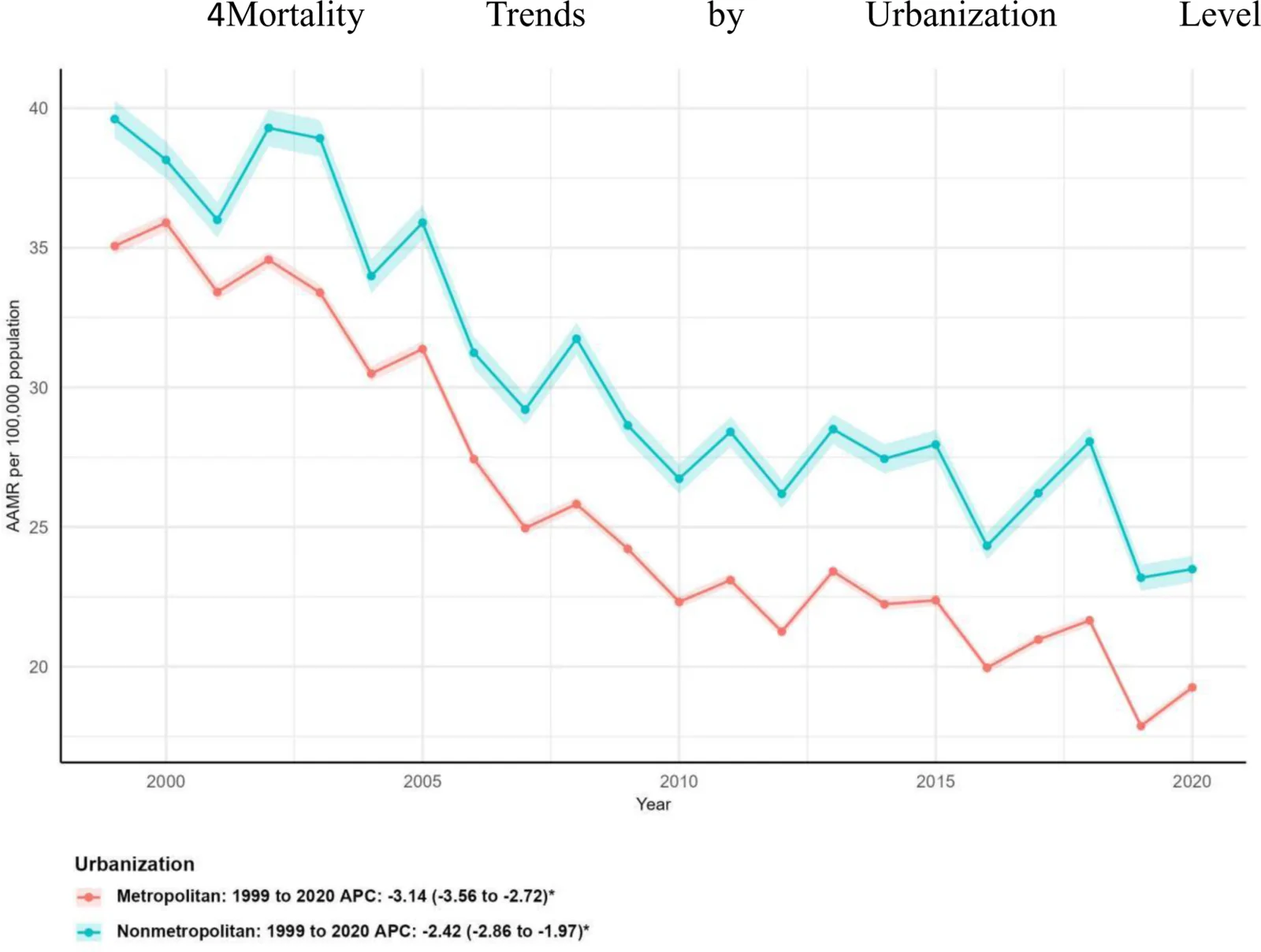
Mortality trends by urbanization level. AAMR = age-adjusted mortality rate, APC = annual percent change.

Throughout the study period, AAMR was consistently higher in nonmetropolitan (rural) areas than in metropolitan (urban) areas. In 1999, the AAMR was 39.61 per 100,000 population for nonmetropolitan areas and 35.06 per 100,000 population for metropolitan areas. In 2020, the AAMR was 23.49 per 100,000 population for nonmetropolitan areas and 19.25 per 100,000 population for metropolitan areas (Fig. 4).

### 3.6. Mortality trends by age group

**Figure 5.**
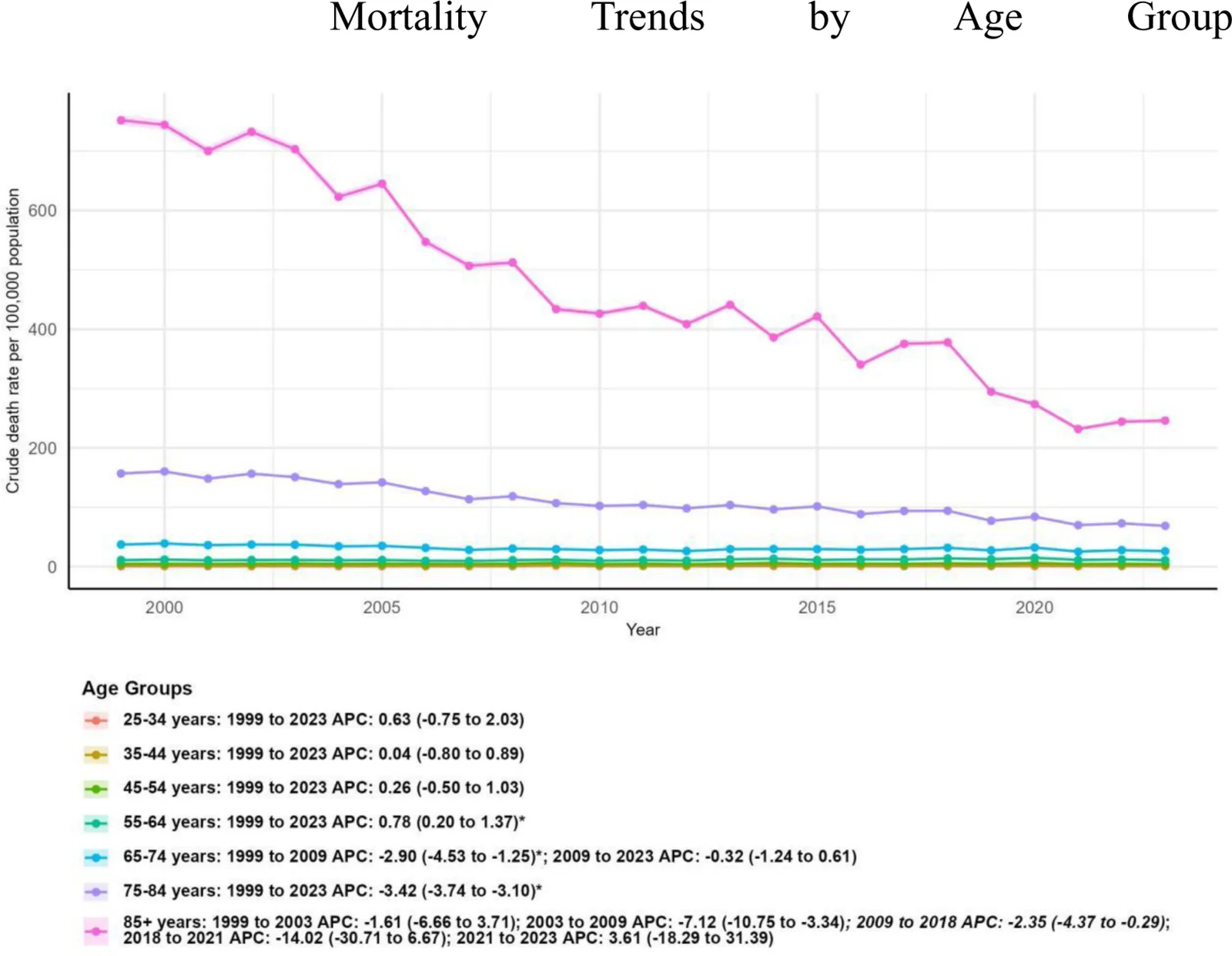
Mortality trends by age group. APC = annual percent change.

In 1999, the crude mortality rate was highest among adults aged 85 years and older (751.78 per 100,000 population) and lowest among those aged 25 to 34 years (0.84 per 100,000 population). In 2023, adults aged 85+ years remained the group with the highest crude mortality rate (245.86 per 100,000 population). The total number of deaths decreased by 51.23% in the 85+ age group, while increasing by 35.10% in the 25 to 34 age group and 75.31% in the 55 to 64 age group (Fig. 5).

## 4. Discussion

This epidemiological analysis tracked mortality risks attributable to influenza and pneumonia over a quarter-century among American adults, relying on ICD-10 J09 to J18 codes to identify fatalities rooted in these 2 respiratory illnesses. Our statistical outputs revealed a steady nationwide fall in age-standardized death metrics, alongside stark health disparities that persisted across demographic and geographic divisions throughout the observation period. These observations align with prior population-based investigations documenting gradual declines in respiratory disease mortality while spotlighting unresolved inequities among distinct population cohorts.^[10,11]^

Gender-based gaps in fatal respiratory outcomes remained consistent across all survey years, with male adults repeatedly facing higher death risks than their female counterparts. This gender divergence has been repeatedly validated in national influenza and pneumonia monitoring datasets released by US public health authorities.^[12,13]^ At the baseline year of 1999, NH Black communities carried the largest baseline mortality burden; although this group experienced the most substantial proportional drop in mortality by 2023, racial disparities had not been fully eliminated by the end of our study window. Such persistent ethnic gaps mirror well-established inequities in infectious disease survival reported in previous population health studies.^[14,15]^ When separating participants by residential setting, rural residents consistently exhibited slower mortality declines and higher overall death rates compared to urban dwellers. This divide echoes widely documented disadvantages in rural medical infrastructure, outpatient accessibility, and community public health outreach for chronic and acute respiratory conditions.^[11,16]^ Among all age brackets, advanced age stood out as the strongest predictor of lethal influenza or pneumonia episodes, consistent with established research confirming extreme vulnerability to severe respiratory complications in adults aged 85 and older.^[10,12]^

One distinctive finding of this study centers on the 55 to 64-year-old middle-aged subgroup, which was the only population segment with a statistically significant rising crude mortality rate across the 25-year timeframe. This emerging risk trend has rarely been highlighted in prior long-duration mortality research, marking a critical signal that public health programs need enhanced targeted surveillance for this middle-aged demographic.^[10,13]^ However, limitations inherent to aggregate ecological data prevent us from pinpointing the exact drivers of this upward shift – potential contributing factors may include generational health differences, updates to death certificate coding standards, shifting comorbidity prevalence, or unmeasured socioeconomic confounders that our dataset cannot capture.

The broad downward trajectory of combined influenza and pneumonia mortality across the United States aligns with nationwide advancements in respiratory disease prevention frameworks and sustained investment in public health infrastructure over recent decades.^[10,17]^ Nevertheless, all interpretations drawn from this observational dataset remain descriptive rather than causal. Our dataset lacks granular individual indicators, including influenza vaccination uptake, inpatient treatment protocols, local medical resource availability, and health policy rollouts, making it impossible to definitively attribute the observed mortality shifts to specific intervention measures.^[18]^

This research advances existing literature in 2 key respects: it extends longitudinal mortality tracking through 2023 to capture post-pandemic shifts following the 2020 COVID-19 crisis, and it delivers full stratified breakdowns across age, gender, ethnicity, and urban–rural residence to unpack layered health inequities. Collectively, our results reinforce the necessity of continuous population-level respiratory disease monitoring to spot newly emerging high-risk groups and tackle long-standing health gaps between distinct community subgroups.

### 4.1. Limitations

This study has key limitations that warrant consideration. A central constraint is that combining influenza and pneumonia using ICD-10 codes J09 to J18 introduces unavoidable misclassification, as the dataset cannot distinguish influenza-associated pneumonia from pneumonia due to other pathogens or separate the independent contributions of each condition.

As an ecological study based on aggregate population-level data, this investigation cannot adjust for individual-level confounders, including comorbidities, health behaviors, socioeconomic status, or vaccination status, and causal inferences cannot be established.

For urban–rural analyses, 2020 data were used as a proxy for 2023 due to database availability, which may introduce uncertainty given changes in rural health care access after 2020. Analyses restricted to underlying causes of death may also not capture the full burden of deaths in which influenza or pneumonia contributed as secondary conditions. Despite these limitations, the study provides reliable, long-term population-level mortality trend and disparity estimates.

## 5. Conclusion

This study demonstrates significant long-term declines in influenza and pneumonia-related mortality among US adults from 1999 to 2023, with persistent disparities by sex, race/ethnicity, age, and urban–rural status. Mortality was highest among adults aged 85 years and older, while a significant increasing trend was observed among adults aged 55 to 64 years. These findings highlight key population-level patterns and inequalities. Further research incorporating individual-level risk factors, vaccination data, and pathogen-specific coding is needed to clarify the drivers of these trends and disparities.

## Author contributions

**Conceptualization:** Nawei Song.

**Data curation:** Nawei Song.

**Formal analysis:** Nawei Song.

**Investigation:** Nawei Song, Juan He, Hana Zhu.

**Methodology:** Nawei Song, Juan He, Xianghua Shuai, Hana Zhu.

**Software:** Juan He, Hana Zhu, Keran Xia.

**Supervision:** Juan He, Hana Zhu, Keran Xia.

**Project administration:** Xianghua Shuai, Hana Zhu.

**Resources:** Xianghua Shuai, Hana Zhu, Keran Xia.

**Validation:** Keran Xia.

**Writing – review & editing:** Nawei Song, Juan He, Keran Xia.
